# Evaluation of full-endoscopic lumbar discectomy in the treatment of obese adolescents with lumbar disc herniation: a retrospective study

**DOI:** 10.1186/s12891-021-04449-5

**Published:** 2021-06-19

**Authors:** Haijiang Yu, Bin Zhu, Qingpeng Song, Xiaoguang Liu

**Affiliations:** 1 Department of Orthopedics, Peking University Third Hospital, Beijing, China; 2 Department of Orthopedics, Capital Medical University Affiliated Beijing Friendship Hospital, Beijing, China

**Keywords:** Adolescent, Obesity, Lumbar disc herniation, Full-endoscopic lumbar discectomy

## Abstract

**Background:**

Obese patients are at risk of complications after spinal surgery. Full-endoscopic lumbar discectomy (FELD) has advantages over conventional open surgery in the treatment of obese adult patients with lumbar disc herniation (LDH) because it can decrease perioperative complications and enhance the degree of patient satisfaction. However, no clinical studies have evaluated the efficacy of FELD in obese adolescents with LDH (ALDH). This study aimed to evaluate the efficacy of FELD for the treatment of obese ALDH.

**Methods:**

We retrospectively collected clinical data from 208 patients with single-segment ALDH who underwent FELD in our hospital between January 2015 and December 2019. According to the WHO classification of obesity, the patients were divided into obese (BMI ≥30 kg/m^2^) and non-obese (BMI < 30 kg/m^2^) groups (control group). Based on the preoperative baseline data of the two groups, propensity score matching was performed to select patients from these groups for a comparative study. Perioperative data included operative time, intraoperative blood loss, and length of postoperative hospitalization. The visual analog scale (VAS), Oswestry disability index (ODI), and modified MacNab criteria were recorded as the main indicators of the surgical outcome. Recurrence rate and incidence of complications were recorded as minor indicators.

**Results:**

Twenty-eight patients and 80 patients were included in the obese and non-obese groups, respectively, after 1:4 propensity score matching. Both groups showed improvements in VAS and ODI scores after surgery and at each follow-up time point (*p* < 0.05). However, there was no significant statistical difference in the surgical outcomes between the two groups at each follow-up time point (*p* > 0.05). The differences in operative time, intraoperative blood loss, and length of postoperative hospitalization were not statistically significant between the two groups (*p* > 0.05).

**Conclusion:**

FELD is a safe and effective minimally invasive technique for treating obese patients with ALDH. The efficacy of FELD in obese and non-obese patients with ALDH was comparable.

## Background

Lumbar disc herniation (LDH) is the most common cause of lower back and leg pain in adults. However, LDH is rare in both children and adolescents. The incidence rate reported in the literature was 0.6–6.8% [1, 2]. However, with improvements in living standards and changes in modern living habits, the incidence of LDH is increasing every year and tends to occur at a younger age [3]. Overweight or obesity is an important risk factor for the onset of adolescent lumbar disc herniation (ALDH), and the proportion of obese patients with ALDH is also increasing [4].

The increasing incidence of obesity has become a serious public health problem worldwide. It was reported that obese adults who undergo spinal surgery have a higher incidence of surgical complications and more surgical blood loss than non-obese patients [5–7]. Compared with traditional open surgery, full-endoscopic lumbar discectomy (FELD), including transforaminal endoscopic lumbar discectomy (TELD) and interlaminar endoscopic lumbar discectomy (IELD), has more advantages, such as less surgical trauma and faster postoperative recovery. Cole et al. [8] believed that its application in obese adults could reduce the length of the surgical incision and the occurrence of complications such as infection, thereby improving clinical efficacy and patient satisfaction. However, no studies have evaluated the short- or mid-term efficacy of FELD in obese adolescents with LDH. Therefore, we conducted a retrospective follow-up study of obese adolescents with LDH who underwent FELD at our hospital for 1 to 5 years. Our study focused on the outcomes and safety of FELD in the treatment of obese patients with ALDH.

## Methods

### Patients

We retrospectively analyzed patients with ALDH who underwent FELD at our hospital between January 2015 and December 2019. The diagnosis of LDH was confirmed based on medical history, physical examination, magnetic resonance imaging (MRI) of the lumbar intervertebral disc, and surgical records. The inclusion criteria included patients with the following: (1) age ≤ 21 years, (2) LDH that was diagnosed by MRI in a single segmental spine, and the clinical manifestations of patients were consistent with imaging findings, (3) no significant improvement of symptoms after 6 weeks of standard conservative treatment, and (4) surgery performed by a senior surgeon (with > 2 years of experience in spinal surgery). The exclusion criteria included patients with the following: (1) lumbar disc herniation in multi-segmental spine; (2) other previous lumbar spine surgeries; (3) spinal fractures, lumbar spondylolisthesis, spinal tumors, scoliosis, or some other orthopedic specialty diseases such as spinal tuberculosis or infection; or (4) severe medical diseases and mental disorders.

A total of 208 patients with ALDH met the above criteria. According to the WHO classification of obesity, these patients were divided into two groups based on body mass index (BMI): obese (BMI ≥ 30 kg/m^2^) and non-obese (BMI < 30 kg/m^2^) groups (control group). To ensure the balance and comparability of the clinical data between the two groups and reduce the confounding bias, propensity score matching (PSM) was performed based on the preoperative baseline data.

### Propensity score matching (PSM)

To reduce the impact of potential confounding factors, the patients in the obese and control groups were matched with propensity scores using all available baseline data. PSM was established through a multivariate logistic regression model, which considered the following variables: age, sex, history of trauma, herniated segment, herniated type, preoperative visual analog scale (VAS) score, and Oswestry disability index (ODI) score. Matching was performed using the nearest-neighbor matching algorithm (caliper width 0.25, standard deviation of the logit score) with a 1:4 ratio without replacement. The use of a 1:4 matching ratio allows for greater statistical power while minimizing the loss of sample size and maintaining matching quality. Standardized mean differences (SMD) were compared before and after PSM to evaluate the matching of balanced potential confounders for the two study groups [9]. Covariates with a standardized difference of < 0.15, in absolute value, were considered satisfactorily balanced.

### Surgical procedure

#### Feld

After the target disc was reconfirmed using the C-arm X-ray fluoroscopy, the puncture point was marked. The puncture needle was sent to the target position, and a guidewire was inserted. A skin incision of approximately 0.8 cm in length was made around the puncture site. The dilating sleeve was then placed along the guidewire step-by-step. Finally, we placed the endoscope after reconfirming the correct position using C-arm X-ray fluoroscopy. Before we formally started the core step of surgery, lumbar discography was performed to identify the fibrous ring crack. The diseased nucleus pulposus tissue was removed using an endoscope, and the dural sac and nerve roots were thoroughly decompressed and loosened until spontaneous pulsation was achieved. Complete hemostasis was achieved with the assistance of bipolar radiofrequency, and the fibrous ring crack was repaired. The endoscope was moved along the nerve root, and the operative field was carefully examined to confirm that no residual compression was present. After endoscope removal, the incision was closed using sutures. The operation was completed without a wound drainage tube.

### Postoperative treatment

In addition to the temporary use of antibiotics during surgery, the patients did not routinely use antibiotics after the operation. The patients were regularly administered symptomatic treatments for nutritional nerves, dehydration, and analgesia. Patients wearing waist circumference were allowed to leave the sickbed 6 h postoperatively. Patients were instructed to rest in bed for 1 month after surgery and to wear waist circumference during daily activities, and to avoid standing for long periods or performing physical labor within 3 months of surgery.

Patients were followed up in the outpatient clinic at 3 months, 6 months, and 1 year after surgery. Subsequently, it was recommended that patients be followed up at least once per year. The re-examination items mainly included computed tomography or magnetic resonance imaging (MRI) of the lumbar spine.

### Clinical evaluation and follow-up

Demographic and perioperative clinical data were obtained by consulting the surgical records and electronic medical records. In addition, we collected data on disease recurrence and complications through telephone and outpatient follow-ups. The demographic data included sex, age, BMI, duration of symptoms, and follow-up time. Perioperative data included operative time, intraoperative blood loss, and length of postoperative hospitalization. The major indicators for evaluating the efficacy of FELD included the VAS score [10] for lower back and leg pain, ODI score [11], and modified MacNab criteria [12] obtained at the last follow-up time point. The minor indicators included the complication and recurrence rates obtained from the medical records or the last follow-up time point. The VAS score was collected before surgery, on the first day after surgery, 3 months after surgery, and at the last follow-up. The evaluation of sexual function was excluded given the sensitive nature of this topic on the adolescent population, and the ODI questionnaire included nine items. In addition, considering that many items in the ODI questionnaire cannot be accurately reflected during the postoperative period in the hospital due to pain or medical advice, the ODI evaluation in this study was only performed before surgery, 3 months after surgery, and at the last follow-up time point. The modified MacNab criteria and its associated clinical efficacy classification were used to evaluate the clinical efficacy of the two groups at the last follow-up. According to the theory proposed by Kraemer et al. [13], we classified the complications of lumbar spine surgery into intraoperative complications such as incorrect level, nerve root lesion, immediate postoperative complications (postoperative complications during hospitalization) such as residual symptoms and new symptoms, and late postoperative complications (complications after discharge). The judgment of recurrence was mainly based on the lumbar MRI diagnosis of the patient after the onset of symptoms or the second surgical treatment.

### Statistical analysis

All data analyses were performed using SPSS version 26.0 (IBM, Armonk, New York, USA). Categorical data are expressed as the number of cases or percentages, and the differences between the two groups were analyzed using the χ^2^ test or Fisher’s exact probability method. Normally distributed measurement data are expressed as the mean ± standard deviation, and Student’s *t*-test was used to compare differences between the two groups. One-way analyses of variance were used to compare VAS or ODI scores within the same group at different times, and the least significant difference method was used for pairwise comparisons. Moreover, measurement data that were not normally distributed were expressed as median and interquartile range and analyzed using the Mann-Whitney U-test to observe differences between the two groups. The linear regression model was applied to assess the relationship between the main indicators of surgical outcome (VAS, ODI, and MacNab classification at the final follow-up time point) and BMI. Differences were considered statistically significant at *P* < 0.05.

## Results

A total of 208 subjects were included in the study, including 37 patients in the obese group and 171 patients in the control group, and 174 patients (83.7%) were followed up. Seven patients (18.9%) and 27 patients (15.8%) were lost to follow-up in the obese and non-obese groups, respectively. However, there was no significant difference in the lost to follow-up rate between the two groups (*p* = 0.641). After excluding patients who were lost to follow-up, there were 30 patients in the obese group and 144 patients in the control group. Finally, 28 and 80 patients were included in the obese and non-obese groups, respectively, after 1:4 PSM. The follow-up time ranged from 12 to 70 months, with a mean follow-up time of 27.75 ± 15.40 months in the obese group and 29.71 ± 16.45 months in the control group. There was no significant difference in follow-up time between the two groups (*p* = 0.582).

The basic preoperative characteristics of each group before and after PSM are shown in Table 1. There were significant differences in the herniated segment, herniated type, and preoperative VAS between the two groups before PSM (*p* < 0.05). However, the balance of each variable was significantly improved after PSM (|SMD| < 0.15, Table 1). The baseline data of the two groups were consistent (*p* > 0.05, Table 1).

**Table 1:** Comparison of preoperative basic characteristics between the two groups before and after 1:4 PSM

	Before PSM				After 1:4 PSM			
Characteristic	Obese (*n* = 30)	Control (*n* = 144)	*p* value	SMD	Obese (*n* = 28)	Control (*n* = 80)	*p* value	SMD
Sex, n (%)			0.074	0.458			0.922	−0.034
Male	26 (86.7)	102 (70.8)			24 (85.7)	66 (82.5)		
Female	4 (13.3)	42 (29.2)			4 (14.3)	14 (17.5)		
Age (years), mean ± SD	18.27 ± 2.26	18.78 ± 2.37	0.275	−0.229	18.07 ± 2.21	18.16 ± 2.61	0.869	−0.058
Duration of symptoms(months), M (IQR)	6 (3.5–12.0)	6 (3.0–12.0)	0.951	0.028	6 (2.5–10.0)	6 (3.0–12.0)	0.916	0.021
History of trauma, n (%)			0.464	−0.157			0.452	−0.095
Yes	6 (20.0)	38 (26.4)			6 (21.4)	23 (28.7)		
No	24 (80.0)	106 (73.6)			22 (78.6)	57 (71.3)		
Herniated segment, n (%)			0.004*	−0.581			0.362	−0.051
L4-L5	23 (76.7)	66 (45.8)			21 (75.0)	50 (62.5)		
L5-S1	6 (20.0)	74 (51.4)			6 (21.4)	28 (35.0)		
Other level	1 (3.3)	4 (2.9)			1 (3.6)	2 (2.5)		
Herniated type, n (%)			0.012*	−0.521			0.280	−0.012
Lateral	20 (66.7)	60 (41.7)			10 (35.7)	38 (47.5)		
Central	10 (33.3)	84 (58.3)			18 (64.3)	42 (52.5)		
Preoperative VAS, mean ± SD	6.20 ± 1.35	6.72 ± 1.06	0.023*	−0.382	6.29 ± 1.36	6.49 ± 1.14	0.444	0.051
Preoperative ODI, mean ± SD	58.47 ± 10.27	61.92 ± 9.22	0.069	−0.366	58.36 ± 10.64	60.36 ± 9.56	0.356	−0.005

*PSM* propensity score matching; *SMD* standardized mean difference; *BMI* body mass index; *VAS* visual analog scale; *ODI* Oswestry disability index;

*SD* standard deviation; *IQR* Interquartile range; *M* Median

*significant difference between two groups

A comparison of preoperative data between the two groups is presented in Table 2. There was no significant difference between the two groups in terms of operative time, intraoperative blood loss, and length of postoperative hospitalization (*p* > 0.05).

**Table 2:** Comparison of perioperative data between the two groups

Parameter	Obese (*n* = 28)	Control (*n* = 80)	*p* value
Operative time(minutes), M (IQR)	50 (42.5 ~ 77.5)	46.5 (34–69)	0.117
Intraoperative blood loss (ml), M (IQR)	5 (5–5)	5 (3.25–5)	0.468
Length of postoperative hospitalization, M (IQR)	3 (2–3)	3 (2–3)	0.337

*IQR* Interquartile range; *M* Median

A comparison of clinical outcome indicators between the two groups is shown in Table 3. The two groups had no significant differences in the main indicators of clinical outcomes, such as VAS scores, ODI scores at each postoperative time point, and the distribution of the MacNab criteria assessments (*p* > 0.05). In accordance with the modified MacNab standard classification, the excellence and good rate of the obese and control groups were 89.3 and 96.3%, respectively, which were also not statistically significant (*p* > 0.05).

**Table 3:** Comparison of the indicators of clinical outcome between the two groups

Parameter	Obese (*n* = 28)	Control (*n* = 80)	*p* value
VAS for lower back and leg pain, mean ± SD			
Preoperative	6.29 ± 1.36	6.49 ± 1.14	0.444
1 day postoperative	1.89 ± 1.20	1.89 ± 0.90	0.980
3 months postoperative	0.79 ± 1.34	0.50 ± 0.76	0.294
Final follow-up	0.50 ± 1.14	0.39 ± 0.75	0.557
ODI(%), mean ± SD			
Preoperative	58.36 ± 10.64	60.36 ± 9.56	0.356
3 months postoperative	8.29 ± 4.97	7.28 ± 2.13	0.305
Final follow-up	8.21 ± 5.30	7.51 ± 2.91	0.386
Modified MacNab, n (%)			0.158
Excellence	22 (78.6)	58 (72.5)	
Good	3 (10.7)	19 (23.8)	
Fair	3 (10.7)	3 (3.8)	
Poor	0 (0)	0 (0)	
Clinical efficacy classification, n (%)			0.365
Excellence and good	25 (89.3)	77 (96.3)	
Fair and poor	3 (10.7)	3 (3.8)	
Complications, n (%)			0.095
Intraoperative	0 (0)	0 (0)	
Immediate postoperative	1 (3.6)	0 (0)	
Late postoperative	7 (25.0)	12 (15.0)	
Recurrence, n (%)			0.259
Yes	1 (3.6)	0 (0)	
No	27 (96.4)	80 (100)	

*VAS* visual analog scale; *ODI* Oswestry disability index; *SD*, standard deviation; *IQR*, Interquartile range; *M* median

**Fig. 1:**
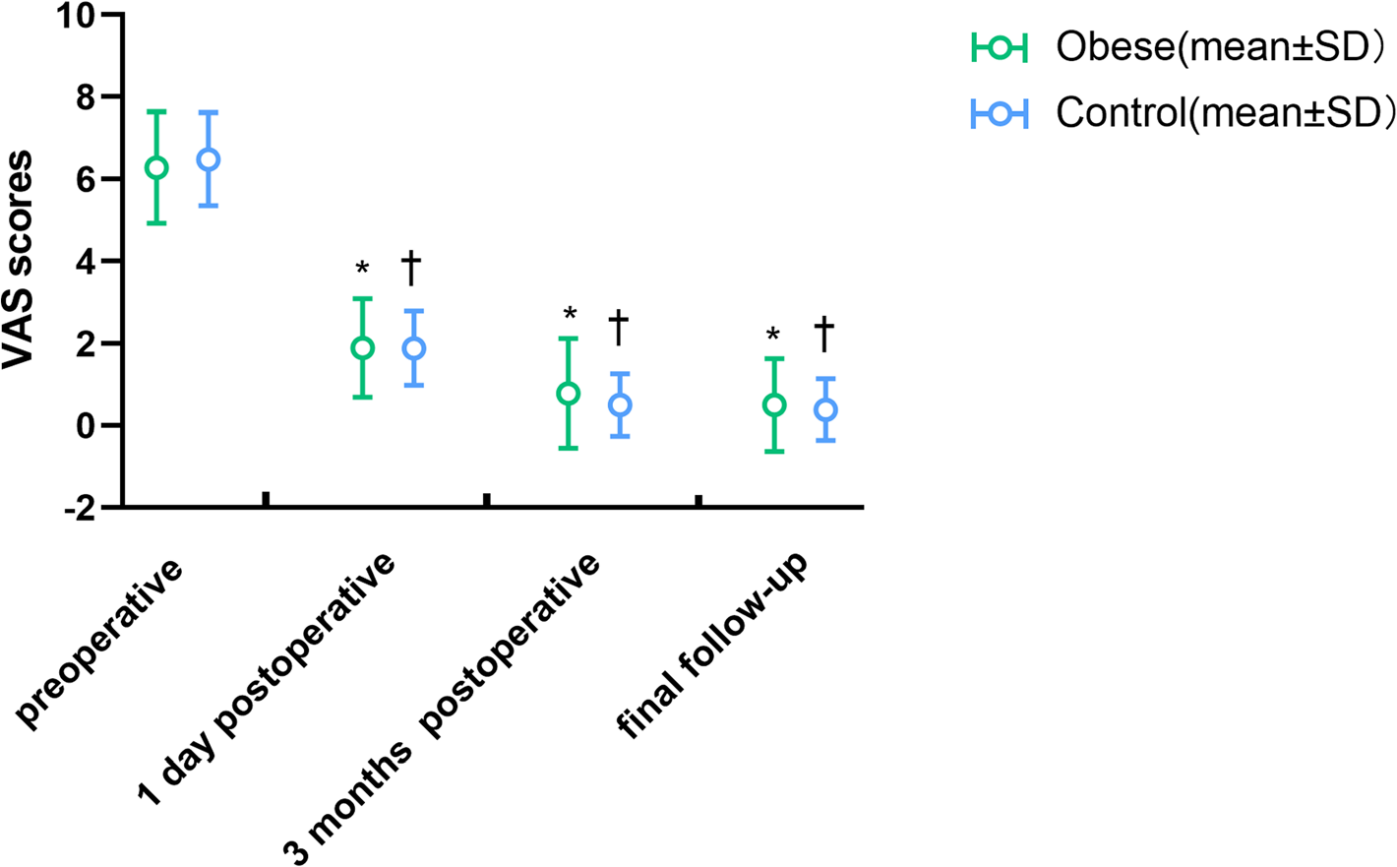
The change of VAS for lower back and leg pain during follow-up. VAS, visual analog scale; SD, standard deviation. *Significant difference between preoperative and postoperative time in the obese group. †Significant difference between the preoperative period and period at each follow-up time in the control group

**Fig. 2:**
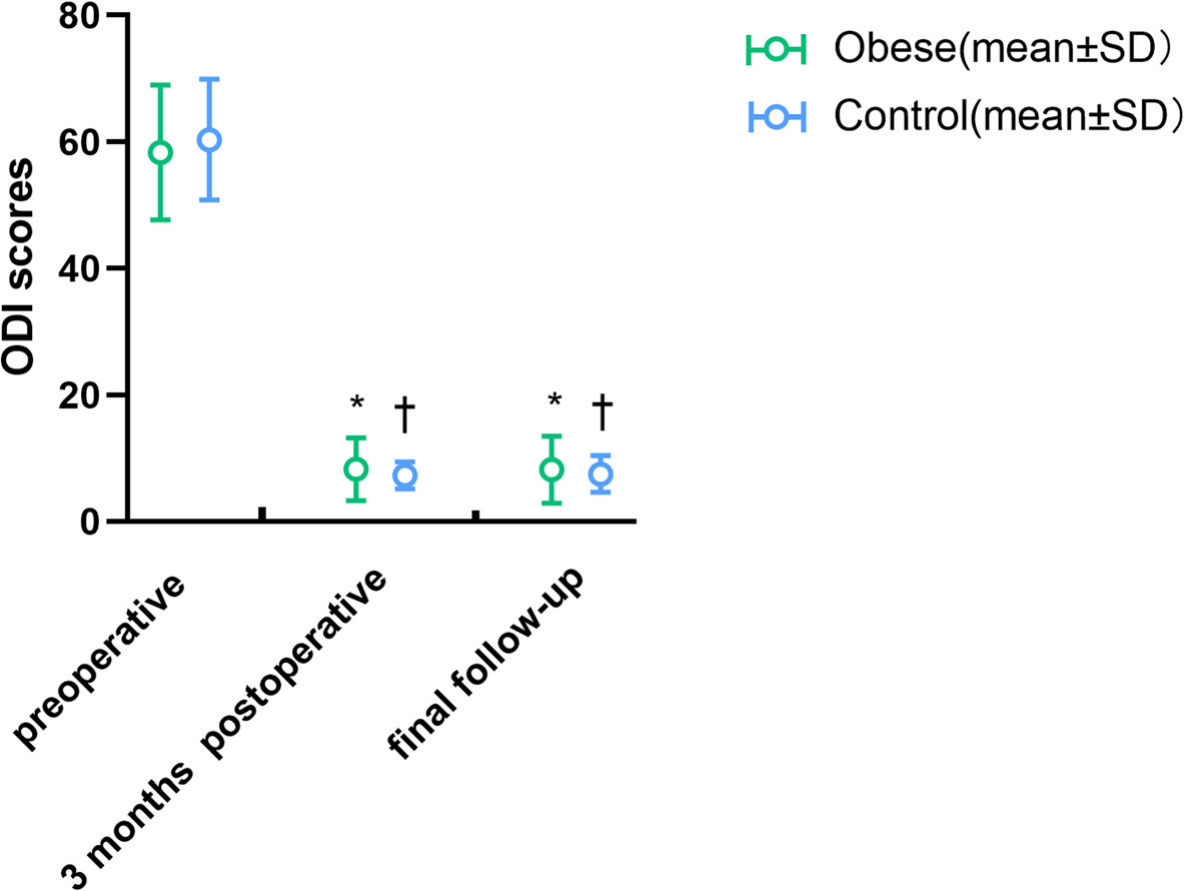
The change of ODI during follow-up. ODI, oswestry disability index; SD, standard deviation. *Significant difference between preoperative and postoperative time in the obese group. †Significant difference between the preoperative period and period at each follow-up time in the control group

In terms of the VAS score changes during the follow-up period (Fig. 1, Table 3), the VAS scores on the first postoperative day, 3 months postoperatively, and at the final follow-up time point were significantly lower than the preoperative VAS scores in both the obese and control groups (*p* < 0.05). Regarding the ODI score changes during the follow-up period (Fig. 2, Table 3), the ODI scores at 3 months postoperatively and at the final follow-up time point were significantly lower than the preoperative VAS scores in both groups (*p* < 0.05). There was no significant difference in the postoperative VAS and ODI scores between the two groups (Fig. 1**,** Fig. 2, and Table 3). In linear regression analysis, the change in BMI was identified as a negative predictor of surgical outcomes (*p*>0.05, Table 4).

**Table 4:** Linear regression analysis of BMI in surgical outcomes

Dependent Variable	B	95% Confidence Interval	*p* value
VAS at final follow-up time point	0.004	−0.028, 0.035	0.810
ODI at final follow-up time point	0.015	−0.118, 0.148	0.825
MacNab classification at final follow-up time point	0.001	−0.021, 0.023	0.956

*VAS* visual analog scale; *ODI* Oswestry disability index

Based on the minor indicators of clinical outcomes of the two groups, there was no significant difference in the overall incidence rate of complications between the two groups (28.6% vs. 15.0%, *p* = 0.112). There were no intraoperative complications in either group. However, one patient had immediate postoperative complications in the obese group. The patient felt that the symptoms of preoperative lower back pain were relieved after returning to the ward, but the pain in the right lower limb was not relieved completely, with the symptom of hyperesthesia on the lateral side of the right lower leg but no neurological symptoms or cauda equina syndrome. Finally, the symptoms gradually resolved 1 year later after he took painkillers and neurotropic drugs intermittently. However, there were no immediate postoperative complications in the control group. Moreover, seven patients had late postoperative complications in the obese group. Six of these patients complained that they felt intermittent pain or discomfort in their lower back or buttocks (VAS scores of 2–3), especially when they were tired, and most of their pain could be relieved without oral analgesics after being sufficiently rested. Only one patient developed more severe pain with no neurological symptoms (VAS scores of 5–6) 1 year after surgery, and this patient went to the local hospital and thus, excluded the possibility of recurrence. Notably, this patient experienced no obvious pain after treatment with Chinese medicine. The conditions in the control group were similar to those in the obese group. Eleven patients in the control group experienced intermittent postoperative mild pain symptoms without oral analgesics, but only one patient required oral analgesics intermittently. In addition, one case (3.6%) in the obese group had a recurrence at the same lumlar disc, and FELD was performed again in our hospital after 1 year postoperatively. However, no recurrence was observed in the control group. And there was also no statistical difference between the two groups in the recurrence rate (*p* > 0.05).

## Discussion

According to the characteristics of the spinal growth and development process, the vertebral epiphyseal cartilage is normally completely fused with the vertebral body around 21 years of age [14]. Hence, 21 years was defined as the upper age limit for patients with ALDH in this study. However, a lower age limit has not been clearly defined. Raghu et al. [15] reported that LDH patients under 12 years of age were very rare, and the youngest patient in this study was a 10-year-old girl.

Over the past 30 years, obesity has become a global epidemic that threatens public health. The prevalence of obesity and overweight increased by 27.5% in adults and 47.1% in children from 1980 to 2013. The number of overweight and obese people increased from 921 million in 1980 to 2.1 billion in 2013 [16]. The prevalence of obesity in children and adolescents has increased significantly. Numerous studies have suggested that obesity is associated with the incidence of various diseases such as cardiovascular disease, cancer, and bone and joint diseases [17–19]. There is evidence that obesity is also an important risk factor for the onset of ALDH [4, 20]. Obesity applies an excessive load to the intervertebral disc, which leads to an abnormal inflammatory response and endocrine regulation in the human body. This eventually results in accelerated degeneration or damage to the intervertebral disc. Obese adolescents accounted for 27.4% of patients with ALDH in our study, so we should closely observe obese people with such diseases whether adults or adolescents.

Spinal surgery in obese individuals is a challenging endeavor for many reasons, including anesthesia, intravenous access, positioning, and wound exposure. Most spine surgeons would agree that surgical intervention is difficult in this population. Before the emergence of minimally invasive spinal surgery, this group of patients usually required longer surgical incisions than the general population to fully expose the surgical area during lumbar disc herniation. However, many complications may occur, including wound infection or poor healing, which affects the surgical satisfaction of these individuals and surgical efficacy [21, 22]. With the development of minimally invasive techniques and the requirement of medical apparatus, FELD has emerged and is widely used in adult and adolescent spinal surgery. Numerous clinical studies have suggested that FELD not only has comparable efficacy to conventional spinal surgery but also has advantages such as reduced blood loss, reduced tissue destruction, and faster postoperative recovery [23–25]. Moreover, several studies [26, 27] have suggested that the application of FELD in obese adults with LDH could reduce the incidence of complications such as wound infection or poor healing. We assume that the decreased incidence of complications in obese patients with FELD can be attributed to technological breakthroughs in the deep surgical field; operations require less time and thus decrease the chances of contamination and par spinal muscle trauma. In addition, FELD is usually performed under local anesthesia; therefore, general anesthesia-related adverse events can be effectively avoided. However, no studies have evaluated the efficacy of FELD in obese patients with ALDH.

Compared with adult patients with LDH, patients with ALDH also have the following characteristics. First, surgical methods were cautiously selected. Regardless of the surgical method used, we need to focus on minimizing the surgical impact on spinal growth and development and the possibility of secondary adjacent segment degeneration or recurrent disc herniation after surgery [28]. Compared with open surgery, FELD minimizes structural damage in the normal spine, such as muscles and facet joints, and reduces the recurrence rate of postoperative iatrogenic instability. Second, the scope of surgical resection is limited. Whether it is FELD or open surgery, the scope of discectomy in adolescents should be controlled to create conditions for the regeneration of intervertebral discs [29] and maximize the retention of the remaining disc function. Finally, it is necessary to consider whether the growth and development of the adolescent spine affect the efficacy of surgery. Gulati et al. [30] believed that the growth of the adolescent spine may affect the efficacy of surgery. However, our study showed that in obese or non-obese patients, the excellent and good rate of surgery was more than 93%. Given that FELD is more complicated than traditional open spinal surgery and that there may be complications such as residual nucleus pulposus, intraspinal hematoma, infection, and more [31], it often requires a longer learning curve to maximize its performance [32].

Based on the basic characteristics of the two groups before surgery, we found that there were no significant differences in age, sex, history of trauma, herniated segment, and type of herniation, the severity of preoperative lower back and leg pain, and ODI scores after PSM. Thus, the comparison between the two groups was more reliable. In terms of perioperative data such as operative time, intraoperative blood loss, and length of postoperative hospitalization, there was no statistical difference between the obese and control groups (Table 3, *p* > 0.05). Therefore, indicating that compared to non-obese patients, obese patients with ALDH may accept FELD without significant difficulties. We believe that FELD causes less surgical trauma and requires no wound drainage tubes. Hence, FELD can effectively shorten the postoperative bedridden recovery time, reduce the occurrence of bed-related complications and hospitalization costs, improve postoperative quality of life, and help patients return to their normal life or work faster. This is in line with the current concept of enhanced recovery after surgery in the field of spinal surgery [33]. However, this was contrary to the results reported in some studies that microdiscectomy or open surgery increased the amount of intraoperative blood loss and length of hospitalization in obese adults [7, 34]. This indicates that FELD is advantageous in the treatment of obese patients with LDH from the other side.

Based on the main indicators of clinical outcome, the postoperative VAS and ODI scores of both groups were significantly lower than the preoperative scores (Figs. 1 and 2, *p* < 0.05), and there was no difference in VAS and ODI scores between the two groups at each follow-up time (Table 3, *p* > 0.05). This indicated that the clinical efficacy of FELD in both obese and non-obese adolescents was comparable and not affected by obesity. Rihn et al. [6] conducted a 4-year follow-up study of 854 non-obese and 336 obese adults and found that obese patients had less improvement of pain symptoms or postoperative ODI scores than non-obese patients. However, the researchers did not clearly explain the surgical method used. Although their results in adults differed from our results in adolescents, it may also suggest that the efficacy of FELD in adolescent patients may not be affected by obesity. From the perspective of minor indicators of clinical outcome, there were no serious complications in either group. The overall incidence of complications was not significantly different between the two groups (Table 3, *p* > 0.05). Moreover, the postoperative complications of FELD in our study mainly manifested as recurring lower back pain or insignificant relief of postoperative pain symptoms, which existed in both the obesity and control groups. However, pain in most patients had little effect on their daily lives or required analgesic intervention. The patient with immediate postoperative leg pain and hyperesthesia in obese group seemed to be postoperative dysesthesia (POD), which was one of the most common postoperative complications associated with FELD. Various reasons, including consistent compression on the exiting root by the working cannula, direct injury by the instrumentation and heat injury caused by radiofrequency, may contribute to exiting nerve injury [35, 36].

It should be pointed out that the recurrence rate in the obese group was similar to the rate in the non-obese group (Table 3, *p* > 0.05), which was also lower than the level (17.57 - 50%) reported in previous studies [37, 38]. However, many studies have shown that obesity was one of the independent significant risk factors for recurrence after FELD [39, 40]. We believe that the following three aspects may explain this difference. First, older age was also one of the risk factors for recurrence [39, 40], so younger patients may have a lower recurrence rate. Second, the number of patients included in the study was small and there was a certain proportion of patients who were lost to follow-up. Furthermore, the follow-up time was not sufficiently long. Third, most adolescents were highly compliant and may adopt stricter postoperative rehabilitation plans under the supervision of their parents.

However, there were some limitations to our study. First, a retrospective, single-center study design was used. Second, the number of patients included and the follow-up time was limited, and there were some patients in whom follow-up data could not be obtained, which lessened the accuracy of the present cohort. Therefore, a prospective randomized controlled trial with larger sample size is needed to confirm our results.

## Conclusion

In summary, this retrospective study suggested that FELD is a safe and effective minimally invasive technique for the treatment of obese patients with ALDH. The improvements in pain and disability in obese patients with ALDH were comparable with those in non-obese patients with ALDH. There was no difference in intraoperative blood loss, operative time, and length of postoperative hospitalization between these groups. Obese patients with ALDH who underwent FELD do not face a greater risk of complications and recurrence, indicating that FELD has good short- and mid-term effects. However, a prospective randomized controlled trial with larger sample size is needed to confirm our results.

## Data Availability

The datasets used and/or analyzed during the current study are available from the corresponding author upon reasonable request.

## Abbreviations

BMI: Body mass index
FELD: Full-endoscopic lumbar discectomy
LDH: Lumbar disc herniation
ALDH: Adolescent with lumbar disc herniation
VAS: Visual analog scale
ODI: Oswestry disability index
SMD: Standardized mean difference
PSM: Propensity score matching
MRI: Magnetic resonance imaging
SD: Standard deviation
IQR: Interquartile range
M: Median
RCT: Randomized controlled trial
