# Correction to: Evaluation of full-endoscopic lumbar discectomy in the treatment of obese adolescents with lumbar disc herniation: a retrospective study

**DOI:** 10.1186/s12891-021-04518-9

**Published:** 2021-08-05

**Authors:** Haijiang Yu, Bin Zhu, Qingpeng Song, Xiaoguang Liu

**Affiliations:** 1 Department of Orthopedics, Peking University Third Hospital, Beijing, China; 2 Department of Orthopedics, Capital Medical University Affiliated Beijing Friendship Hospital, Beijing, China

**Correction to: BMC Musculoskelet Disord 22, 562 (2021)**

**https://doi.org/10.1186/s12891-021-04449-5**

Following the publication of the original article [1] the authors noticed an introduced error in the authors’ footnote during production process. The last author name is incorrect.

The published authors’ footnote was “† Haijiang Yu, Bin Zhu and Xiaoguang Liu are contributed to the work equally and should be regarded as co-first authors.” it should be “† Haijiang Yu, Bin Zhu and Qingpeng Song are contributed to the work equally and should be regarded as co-first authors.”

The original article [1] has been updated.
